# Reliability of Ultrasound Muscle Assessment in People With Critical Illness Performed by Occupational Therapy Students and Occupational Therapists

**DOI:** 10.1155/oti/5525893

**Published:** 2026-03-18

**Authors:** Felipe Douglas Silva Barbosa, Raquel Pereira de Santana, Maria Isabel Souza Matos, Alana Rios Garcia Santos

**Affiliations:** ^1^ Department of Family Health and Occupational Therapy, Faculty of Medicine, Federal University of Bahia (UFBA), Salvador, Bahia, Brazil, ufba.br; ^2^ Department of Rehabilitation, University Hospital Professor Edgard Santos, Federal University of Bahia (UFBA), Salvador, Bahia, Brazil, ufba.br

**Keywords:** intensive care unit, muscles, occupational therapy, test reproducibility, ultrasonography

## Abstract

The use of ultrasound as an assessment tool by occupational therapists in the context of intensive care can support the clinical reasoning in identifying the impacts of musculoskeletal alterations on the ability to perform activities of daily living and other meaningful occupations. However, ultrasonography is not part of the core curriculum in occupational therapy education, and the knowledge and technical requirements necessary for its use by these professionals remain unclear. Therefore, the aim of this study was to analyze the intraexaminer and interexaminer reliability of ultrasound muscle assessment among occupational therapy students and occupational therapists after the completion of a standardized training program. Data collection included 8 h of examiner training and image acquisition. Three examiners were trained by an occupational therapist experienced in muscle assessment using ultrasound, including two occupational therapy undergraduate students and one occupational therapist. Each examiner performed two assessments of each muscle group, without knowing the results of their own evaluations or those of the other examiners. Participants were recruited by convenience, including adults and older people who were admitted to the intensive care unit. The sample size of this study was seven participants. For descriptive analyses, categorical variables were expressed in absolute and relative frequency. Continuous variables were presented as median and interquartile range or as mean and standard deviation. For the interobserver and intraobserver reliability analyses, the values of the intraclass correlation coefficient were performed. A total of 280 US images were analyzed. In regards to inter‐examiner reliability, excellent results were found for all muscle groups (ICC: 0.785–0.986). For the intraexaminer reliability, excellent results were found in the majority of analyzes performed (ICC: 0.779–0.997), with two exceptions. Occupational therapy students and occupational therapists demonstrated excellent reliability in the ultrasound assessment of muscle thickness. These findings may support the clinical reasoning of occupational therapists to improve outcomes in occupational performance and participation of critically ill survivors.

## 1. Introduction

Ultrasound (US) has been used as a method for both quantitative and qualitative assessment of muscle structure in people in intensive care units (ICUs) [1]. This assessment has been applied while the person is not able to cooperate with volitional functional tests [2]. Thus, ultrasonographic assessment of muscle changes has been suggested as a prognostic marker, being associated with functional outcomes in people with critical illnesses, such as muscle strength and the ability to ambulate and perform activities of daily living, which directly impact quality of life [2–4].

However, the relationship between the capacity to perform activities of daily living and muscle changes observed through ultrasonography remains underexplored. In a recent systematic review with meta‐analysis, Barbosa et al. [3] reported that only two studies investigated this association, which did not allow for its analysis through meta‐analysis. In this regard, there is a need for further investigation of this relationship, and occupational therapy is a profession with a particular interest in phenomena related to individuals′ capacity and participation in activities of daily living and other meaningful occupations.

The use of US by occupational therapists is a recent topic of discussion, especially with the expansion of point‐of‐care ultrasound (POCUS) to other health professions, with emphasis on its use within the scope of occupational therapy, as a tool integrated into occupation‐centered practice [5]. Within their professional scope, occupational therapists seek to understand clients′ occupational needs, which includes comprehending the multiple factors that affect performance and participation, as well as the elements that can support intervention processes. These include aspects of the person, the occupation, and the context, as well as their interrelationships. In this sense, the use of US by occupational therapists is related to the assessment of body structures and functions, components of the person, which, together with other information, support professional reasoning, therapeutic planning, and the analysis of achieved outcomes [6, 7].

Roll [8] and Roll et al. [8] report evidence that such resources can support clinical decision‐making, serve as complementary tools in intervention processes, and assist in the education of professionals, students, and people assisted, particularly in the context of physical rehabilitation. However, the literature indicates greater use in hand therapy and upper‐limb rehabilitation, with a lack of studies in other areas, such as acute care. In regards to acute care, Occupational therapists have been engaged in ICUs to enhance occupational performance during and after critical care, implementing interventions targeting activities of daily living, mobility, and motor function rehabilitation, with ICU‐acquired weakness identified as a key contributor to deficits in this performance. [9] Therefore, there is plausibility for the use of ultrasonography by occupational therapists, considering the high prevalence of limitations in activities of daily living and ICU–acquired muscle weakness, as well as the association between these factors as demonstrated by other assessment methods [10].

However, since this is a resource that has only recently been discussed in the profession, it is believed that US is not part of the standard training in undergraduate occupational therapy programs, and access to such training is possible through postgraduate courses, when available [5]. Moreover, no studies were found reporting the training of occupational therapists and occupational therapy students in muscle assessment using US, which has already been investigated in other professional categories, such as physical therapy [11, 12]. Thus, the training needs of occupational therapy students and occupational therapists for the use of musculoskeletal ultrasonography in people with critical illnesses are not yet known.

Therefore, the aim of this study is to analyze the intraexaminer and interexaminer reliability of muscle assessment using US among occupational therapy students and occupational therapists after the completion of a standardized training program.

## 2. Materials and Methods

### 2.1. Study Design, Setting, and Ethical Considerations

This research was conducted to investigate the interexaminer and intraexaminer reliability of US muscle assessment. It was conducted in a general ICU of a university hospital in Brazil. In regards to ethical considerations, this study was approved by the Research Ethics Committees of the Federal Univeristy of Bahia and University Hospital Professor Edgard Santos (Approval: 78406424.2.3001.0049). In addition, this research followed national and international regulations and all participants and caregivers were informed about the study conditions, guarantee of anonymity and the possibility of withdrawing from the research at any time, without any prejudice. After this process, they signed the “informed consent form.” Finally, this study followed the guidelines for reporting reliability and agreement studies (GRRAS) [13] and the consensus‐based standards for the selection of health measurement instruments (COSMIN) risk of bias tool to assess the quality of studies on reliability and measurement error of outcome measurement instruments [14].

### 2.2. Population, Sample, Inclusion, and Exclusion Criteria

Participants were recruited by convenience, including adults and older people (≥ 18 years old) who were admitted to the ICU. The sample size calculation was done following the recommendations from Walter et al. [15], including the acceptable reliability values of 0.60, the predicted reliability coefficient of 0.94 [16], four raters, two repetitions of the task, and levels of error Types I (*α* = 0.05) and II (*β* = 0.20). Thus, six participants were required as sample, but we recruited seven participants to reduce losses. These parameters were also tested according to Bonett [17], which resulted in a sample of two participants, so we have decided to maintain seven participants. All sample analyses were performed via Sample Size Calculator 2.0 [18].

Possible participants were analyzed through the inclusion and exclusion criteria, including adults and older people who were already admitted to the ICU at the time of the data collection initiation, as well as participants who were admitted later, until the required sample was reached. Participants were excluded if: (1) They had a diagnosis of dementia, terminal cancer, were in palliative care, or received home mechanical ventilation before admission; (2) they remained hospitalized for less than 48 h; (3) they had a second or subsequent admission to the ICU during the study period; and (4) they did not have family members or caregivers who could give information or sign the “informed consent form.”

### 2.3. Data Collection

Data collection included examiner training and image acquisition, conducted between September and November 2024. Four examiners were involved in the data collection: one occupational therapist experienced in muscle assessment using US, PhD and university lecturer, and three novice examiners: two undergraduate students in occupational therapy and one occupational therapist with 22 years of professional experience and 10 years working in an ICU. The three novice examiners were trained by the expert examiner. The training followed the protocol described by Barbosa et al. [16], consisting of 8 h of instruction and covering the following content: participant positioning, equipment usage, image acquisition, and analysis of muscle thickness.

In summary, after training, the four evaluators acquired two images each to analyze interexaminer and intraexaminer reliability. Participants were examined in the supine position, semireclined at a 30° angle, with limbs extended and muscles relaxed. US images were obtained on the right side of the body, using US gel to ensure adequate acoustic coupling, with minimal pressure applied to the participant′s skin. Examiners alternated following each image acquisition in an interval of up to 24 h between assessments to reduce measurement variability. To ensure independent image acquisition and minimize bias, participants were returned to their initial position after each attempt, the skin was cleaned, and the US device was reset to a new file for each evaluator. In addition, evaluators had no access to their images and measurements, and all images were stored on an unidentified hard drive [16].

For image acquisition, the evaluation focused on five muscles/muscle groups: biceps brachii, forearm flexor group, quadriceps femoris, tibialis anterior, and diaphragm. The protocol defined the exact location for transducer placement and the anatomical structures to be visualized in the image (such as bone and fascia) to identify the targeted muscle. For the biceps brachii, the transducer was placed on the anterior surface of the arm, at the proximal two‐thirds of the distance between the acromion and the cubital fossa. Muscle thickness was measured as the distance between the humerus and the superficial fascia of the biceps brachii, including the brachialis muscle. For the forearm flexor muscles, the transducer was positioned on the anterior surface of the forearm, at the proximal two‐fifths of the distance between the antecubital crease and the distal end of the radius. Muscle thickness was measured between the interosseous membrane, adjacent to the radius, and the superficial fascia of the flexor muscles. Regarding the quadriceps femoris, the transducer was placed on the anterior surface of the thigh, at the proximal two‐thirds of the distance between the anterior superior iliac spine and the superior border of the patella. Muscle thickness was determined as the distance between the femur and the superficial fascia of the rectus femoris, including both the rectus femoris and the vastus intermedius muscles. For the tibialis anterior, the transducer was positioned on the anterior surface of the leg, at the proximal one‐quarter of the distance between the inferior border of the patella and the tip of the lateral malleolus. Muscle thickness was measured between the interosseous membrane, adjacent to the tibia, and the superficial fascia of the tibialis anterior. Finally, for the diaphragm, the transducer was positioned at the eighth or ninth intercostal space, between the anterior and midaxillary lines, corresponding to the zone of apposition. Diaphragm thickness was measured as the distance between the pleural and peritoneal lines, not including the lines, at the end of expiration. Participants who agreed to take part in the study and signed the informed consent form were recruited [16, 19–21]. Each assessment took approximately 10 min per examiner.

The training and assessments were conducted using a LOGIC e R‐7 US device (GE Medical System Co. Ltd, China), equipped with a high‐frequency linear array transducer (Model 12L‐RS), in B‐mode. All procedures followed the protocol previously established by Barbosa et al. [16]. A guiding document was also provided to be used during image acquisition.

### 2.4. Statistical Analysis

For descriptive analyses, categorical variables were expressed in absolute and relative (percentage) frequency. Continuous variables were submitted to Shapiro–Wilk test to determine the normality of the distribution. Although no violation of normality was observed (*p* > 0.05), considering the small sample size (*n* = 7), the data are presented as median and interquartile range. In regards of muscle thickness, the averages of the two assessments performed on each muscle group per examiner were presented as means and standard deviations. For the interobserver and intraobserver reliability analyses, the values of the intraclass correlation coefficient (ICC) were performed, considering a two‐way mixed‐effects method with absolute agreement. In regards to the interexaminer reliability analysis for each muscle, analyzes were performed using the mean thickness measurement of the two images captured. For intraexaminer reliability, analyzes were performed using the results of the two images of each evaluator for each muscle. To analyze the ICC of each correlation, parameters established by Cicchetti [22] were utilized. A weak correlation was considered when a value lower than 0.4 was found; satisfactory correlation with a value greater than or equal to 0.4 and less than 0.60; a good correlation with a value equal to or greater than 0.60 and less than 0.75; an excellent correlation with a value greater than or equal to 0.75 [22]. All analyzes were performed using software Jamovi, Version 2.7.5., with *p* ≤ 0.05 and a 95% confidence interval (CI).

## 3. Results

As seven participants were included in this study, 280 US images were analyzed. Sample characteristics are presented at Table 1. In general, the majority of the sample was composed of females (57.1%), with age varying between 25 and 71 years old, with a mean age of 51.1 years old. In regards to health condition, most of them had a mean stay of 9.1 days since the admission ICU on the day of the US assessment, 57.1% had a clinical condition upon admission to the ICU, 71.4% did not use mechanical ventilation during their ICU stay, and all of them were awake, without sedation and mechanical ventilation at the time of assessment.

**Table 1:** Participants′ sociodemographic and clinical characteristics.

Characteristics	Results (*n* = 7)
Age in years (median—IQR)	60.0(33.5–67.5)
Sex	
Female (%)	57.1%
Male (%)	42.9%
Condition for admission to the ICU	
Clinical (%)	57.1%
Surgical (%)	42.9%
Days of ICU stay at the time of assessment (Median—IQR)	10.0 (04.5–12.0)
Use of mechanical ventilation during ICU stay	
Yes (%)	28.6%
No (%)	71.4%

In regards of muscle thickness, the averages of the two assessments performed on each muscle group per examiner were presented as means and standard deviations on Table 2. It shows the results of the examined muscles, which included biceps brachii, forearm flexor group, quadriceps femoris, tibialis anterior and diaphragm.

**Table 2:** Mean and standard deviation of muscle thickness per examiner.

	Examiners	*M* *e* *a* *n* (±*S* *D*) in cm
Biceps brachii	Examiner 1 Examiner 2 Examiner 3 Examiner 4	2.40 (±0.36) 2.18 (±0.45) 2.01 (±0.41) 2.07 (±0.29)
Forearm flexor group	Examiner 1 Examiner 2 Examiner 3 Examiner 4	2.20 (±0.35) 2.17 (±0.44) 2.12 (±0.40) 2.12 (±0.28)
Quadriceps femoris	Examiner 1 Examiner 2 Examiner 3 Examiner 4	2.02 (±0.85) 2.10 (±0.89) 2.23 (±0.86) 1.94 (±0.80)
Tibialis anterior	Examiner 1 Examiner 2 Examiner 3 Examiner 4	1.73 (±0.40) 1.78 (±0.27) 1.84 (±0.38) 1.67 (±0.34)
Diaphragm	Examiner 1 Examiner 2 Examiner 3 Examiner 4	0.17 (±0.03) 0.16 (±0.04) 0.17 (±0.03) 0.15 (±0.03)

In regards to muscle thickness intra‐rater reliability, analyzes were performed, which are presented in Table 3. For the intraexaminer reliability, excellent results were found in the majority of analyzes performed (ICC: 0.779–0.997), with the exception of “satisfactory” result for biceps brachii from Examiner 4 and a “good” result for forearm flexor group from Examiner 2. For interexaminer reliability, excellent results were found for all muscle groups (ICC: 0.785–0.986).

**Table 3:** Intraexaminer and interexaminer reliability of muscle thickness according to muscle group.

Muscles	Intraexaminer reliability			Interexaminer reliability	
	Examiners	Results	Correlation	Results	Correlation
Biceps brachii	Examiner 1 Examiner 2 Examiner 3 Examiner 4	0.879 0.921 0.948 0.583	Excellent Excellent Excellent Satisfactory	0.827	Excellent
Forearm flexor group	Examiner 1 Examiner 2 Examiner 3 Examiner 4	0.958 0.748 0.929 0.779	Excellent Good Excellent Excellent	0.785	Excellent
Quadriceps femoris	Examiner 1 Examiner 2 Examiner 3 Examiner 4	0.909 0.997 0.988 0.985	Excellent Excellent Excellent Excellent	0.986	Excellent
Tibialis anterior	Examiner 1 Examiner 2 Examiner 3 Examiner 4	0.971 0.886 0.971 0.936	Excellent Excellent Excellent Excellent	0.950	Excellent
Diaphragm	Examiner 1 Examiner 2 Examiner 3 Examiner 4	0.847 0.913 0.985 0.922	Excellent Excellent Excellent Excellent	0.924	Excellent

## 4. Discussion

In general, the results demonstrated excellent interexaminer and intraexaminer reliability of muscle thickness of biceps brachii, forearm flexors groups, quadriceps femoris, tibialis anterior, and diaphragm of people with critical illness among occupational therapy students and occupational therapists. Firstly, it is important to highlight that this study expands the results of a prior study [16], demonstrating that 8 h of training can be enough to enable novice examiners to perform US muscle assessment.

When comparing the reliability results of this study with others, Mayer, Dhar, et al. [12] reported similar findings. That study analyzed the reliability of the assessment of the biceps brachii, quadriceps femoris, and tibialis anterior muscles following an 8‐h training session. Regarding muscle thickness, interexaminer reliability was excellent (ICC: 0.886–0.940). However, Mayer, Dhar, et al. [12] did not report intraexaminer reliability data. Other studies also demonstrated excellent interexaminer and intraexaminer reliability of novice examiners [11, 23].

A different approach was done in a study done by González‐Seguel et al. [24]. They had a 20‐h program, which included 6 h of online classes and an in‐person course, with theorical classes and practical training. Reliability showed moderate to excellent results in regards to landmarking and muscle thickness measurements, but they were absent to weak in regards to vastus lateralis pennation angle. Also, participants had an 89.1% score on theorical questionnaire assessment [24]. The same questionnaire was utilized in other study, and demonstrated that 6.5 h of training was not enough to improve theorical knowledge on diagnostic lung and lower limb US [25].

It is observed in the study by Mayer, Dhar, et al. [12] and others [26, 27] that lower limb muscles, particularly the quadriceps and tibialis anterior, are frequently assessed in the literature, showing excellent reliability in several studies. Our study also demonstrated excellent reliability for these analyses. On the other hand, upper limb muscles are less frequently evaluated compared with these muscles. Our results corroborate the findings in the literature regarding interexaminer reliability for the biceps brachii ([12, 16, 23] and the forearm flexors group [28]. Regarding the forearm flexor group, a similar result was also observed in a previous study [16]. That study reported that the need for positioning adjustments may have influenced the findings [16].

Finally, it is important to highlight that this is the first study done that included only occupational therapy students and occupational therapists performing US muscle assessment of this population. This represents an important advancement for occupational therapy, as it may serve as a direct method for assessing muscle conditions that may impact, or are already impacting, the occupational performance and participation of people who survive critical illness. Another important aspect is that most studies assess only a single muscle or muscle group, primarily the quadriceps femoris. However, there are no studies demonstrating that changes in a single muscle or muscle group reflect whole‐body changes or that they are related to changes in the capacity to perform activities of daily living and other occupations [3]. Thus, this study expands the possibilities for analyzing this relationship, considering that multiple muscles may be involved in the performance of occupations.

This study has some limitations. First, its observational design, particularly the temporal nature of the measurements, does not allow control over potential confounding factors and limits the ability to infer causal relationships. Second, the small sample size can reduce the precision of reliability estimates. Third, although both intraexaminer and interexaminer reliability were assessed, other clinimetric properties, such as responsiveness and validity, were not evaluated. Additionally, other assessment approaches could have been used as part of the training standardization, such as knowledge and training satisfaction questionnaires, which could have strengthened the findings. Therefore, these results should be interpreted with caution.

## 5. Conclusion

This study is aimed at analyzing the intraexaminer and interexaminer reliability of muscle assessment using US among occupational therapy students and occupational therapists after the completion of an 8‐h standardized training program. Occupational therapy students and occupational therapists demonstrated excellent reliability in the US assessment of muscle thickness in the biceps brachii, forearm flexor group, quadriceps femoris, tibialis anterior, and diaphragm of people with critical illness. This result suggests that a standardized training program can provide the target group with the knowledge and skills necessary to implement the established protocol.

## Author Contributions

All named authors meet authorship criteria. **Felipe Douglas Silva Barbosa**: conceptualization, methodology writing—review & editing, formal analysis, investigation, data curation, writing—original draft, writing—review & editing, project administration. **Raquel Pereira de Santana**: conceptualization, methodology, investigation, data curation, writing—original draft. **Maria Isabel Souza Matos**: conceptualization, methodology writing—review & editing, investigation, data curation, writing—original draft. **Alana Rios Garcia Santos**: conceptualization, methodology writing—review & editing, investigation, data curation, writing—original draft.

## Funding

No funding was received for this manuscript.

## Conflicts of Interest

The authors declare no conflicts of interest.

## Data Availability

The data that support the findings of this study are available from the corresponding author upon reasonable request.
